# Registration between Pathological Image and MR Image for Comparing Different Modality Images of Brain Tumor

**DOI:** 10.1155/2014/430762

**Published:** 2014-12-08

**Authors:** Yuka Nakamura, Takuya Tanaka, Takashi Ohnishi, Noriaki Hashimoto, Hideaki Haneishi, Jennie Taylor, Matija Snuderl, Yukako Yagi

**Affiliations:** ^1^Graduate School of Engineering, Chiba University, Chiba 263-8522, Japan; ^2^Department of Medical System Engineering, Chiba University, Chiba 263-8522, Japan; ^3^Center for Frontier Medical Engineering, Chiba University, Chiba 263-8522, Japan; ^4^Massachusetts General Hospital, Boston, MA 02114, USA; ^5^New York University Langone Medical Center, New York, NY 10016, USA; ^6^Massachusetts General Hospital Pathology Imaging and Communication Technology (PICT) Center, Boston, MA 02114, USA; ^7^Harvard Medical School, Boston, MA 02215, USA

## Background

Magnetic resonance imaging (MRI) is a preferred modality for brain tumor detection and preoperative localization. However, invasive regions of tumor are often unclear in MR image and thus it is difficult to identify tumor from MR image. Therefore, for revealing the relationship between cells and genetic information of the tumor of MR image, it is required to compare pathological images with MR images on the same regions. In this study, we deal with glioblastoma among brain tumors. However, it would be difficult to compare them directly because pathological images are deformed through tissue specimen making. Thus, registration from MR image to pathological image is performed through the macroimage of cross section which is taken before cutting the brain into blocks. Pathological image to be compared with MR image is combined from multiple histology images and deformed by referring the macroimage. The detail of this technique is described in another paper submitted to this conference IADP2014. In this paper, we assume to have obtained such a pathological image. It is necessary to search appropriate curved plane from MR image because macroimage to be referred in merging pathological images has a possibility to be extracted as a curved plane from brain.

## Method

**Figure 1:**
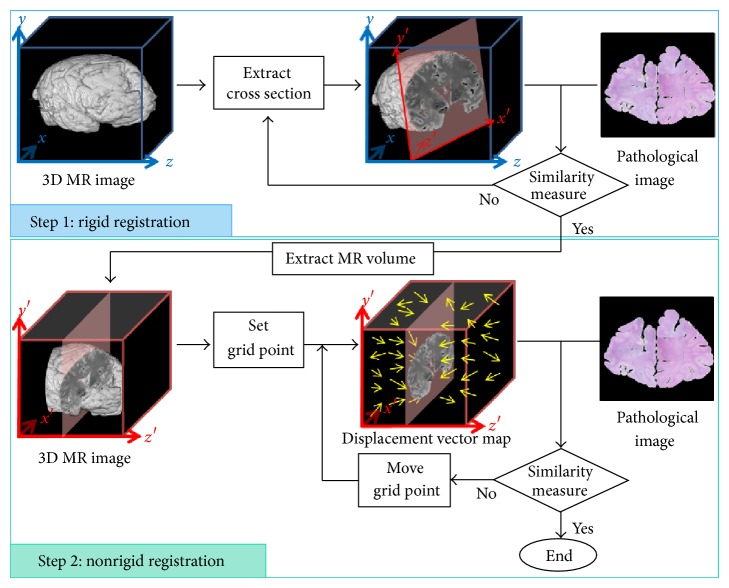
Flow of method.

We propose a two-step registration of pathological image and MR image. The flow of our proposed method is described in Figure 1. In the first step, 3D MR image is roughly registered by a rigid registration algorithm and corrected position, where the 2D image in a predetermined cross section of the volume data is registered to the reference pathological image. Conditional mutual information (CMI) is used as a similarity measure and the optimization is performed by artificial bee colony algorithm (ABCA). In the second step, 3D MR image is precisely registered by a nonrigid registration and the corrected distortion that is not considered in step 1. Displacement vectors are prepared at the nodes arranged on the 3D grid on the rigid-transformed 3D MR image, and the 2D image in a predetermined cross section of the distorted volume data is compared with the reference pathological image. For optimization, we employed a combined measure which consists of three evaluation values: CMI, the overlapped region between the extracted 2D MR image and the reference pathological image, and the local smoothness of displacement vector variation. The objective function is minimized by ABCA and Nelder-Mead simplex algorithm.

## Results

**Figure 2:**
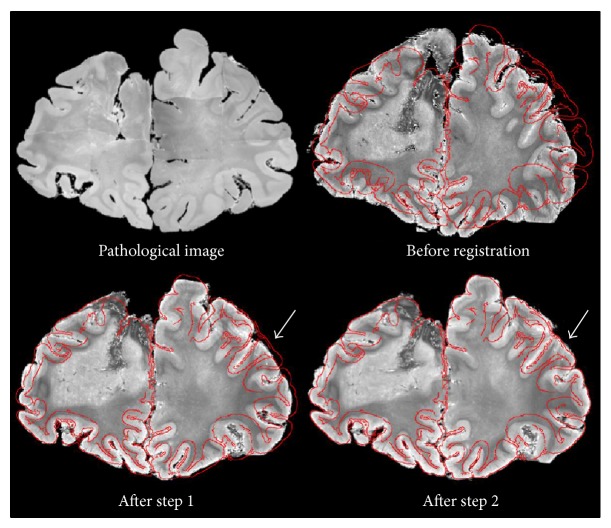
Results of each step.

The results of these steps are shown in Figure 2. We could correct MR position accurately by step 1, and MR cross section similar to the pathological image was extracted. From the result of step 2, we can see the MR cross section that was deformed correctly, especially in the arrowed region. We also confirmed that the inner pattern of the MR cross section had a resemblance to that of the pathological image.

## Conclusion

We developed a two-step registration method of pathological image and MR image. Through experiments, we obtained similar cross section to pathological image from MR image.

